# Mouse TMCO5 is localized to the manchette microtubules involved in vesicle transfer in the elongating spermatids

**DOI:** 10.1371/journal.pone.0220917

**Published:** 2019-08-08

**Authors:** Kenya Yamase, Yoko Tanigawa, Yasufumi Yamamoto, Hiromitsu Tanaka, Tohru Komiya

**Affiliations:** 1 Department of Biological Function, Graduate School of Science, Osaka City University, Sugimoto, Sumiyoshi, Osaka, Japan; 2 Faculty of Pharmaceutical Sciences, Nagasaki International University, Sasebo, Nagasaki, Japan; University of Minnesota Medical School, UNITED STATES

## Abstract

As a result of a high-throughput *in situ* hybridization screening for adult mouse testes, we found that the mRNA for *Tmco5* is expressed in round and elongating spermatids. *Tmco5* belongs to the *Tmco* (*Transmembrane and coiled-coil domains*) *gene family* and has a coiled-coil domain in the N-terminal and a transmembrane domain in the C-terminal region. A monoclonal antibody raised against recombinant TMCO5 revealed that the protein is expressed exclusively in the elongating spermatids of step 9 to 12 and is localized to the manchette, a transiently emerging construction, which predominantly consists of cytoskeleton microtubules and actin filaments. This structure serves in the transport of Golgi-derived non-acrosomal vesicles. Moreover, induced expression of TMCO5 in CHO cells resulted in the co-localization of TMCO5 with β-tubulin besides the reorganization of the Golgi apparatus. Judging from the results and considering the domain structure of TMCO5, we assume that *Tmco5* may have a role in vesicle transport along the manchette.

## Introduction

Gametes play critical roles in inheriting genetic information from generation to generation. Above all, sperms are specially organized vehicles to convey and pass the information to eggs. Studies focused on the mode of this succession of the genome have been a major field of developmental biology and medicine [1].

Spermatogenesis occurs in seminiferous tubules of adult testes, in which spermatogonia, the male germline stem cells, give rise to spermatocytes, spermatids, and finally to immature spermatozoa. After moving into epididymis, the spermatozoa undergo functional maturation via exposure to an acidic microenvironment and a variety of secretory proteins in the organ [2].

During spermiogenesis, the final stage of the spermatogenesis, spermatids that have completed meiosis differentiate into spermatozoa with several well-defined reactions. Firstly, condensation of chromatin is caused by changing chromatin binding proteins from histones to transition proteins and finally to protamine [3]. Secondly, drastic morphological changes occur by developing cytoskeleton systems to create novel structures [4]. Thirdly, in order to acquire the ability of movement, energy-producing mitochondria are densely packed to the midpiece [5], and a flagellum is constructed to the tail. Fourthly, the functions required for fertilization are equipped; acrosome for passing the zona pellucida [6], proteins for cell membrane fusion [7, 8], and protein kinases for activating eggs [9, 10]. Increasing the information about the function of the genes required for each step is essential for understanding the molecular mechanism of spermiogenesis along with finding fertility treatment.

Previously, we isolated several genes that are specifically expressed in the intestinal and epidermal tissues of mice [11–15] by using a high-throughput *in situ* hybridization system, wherein almost all the procedures were carried out by 96-well format. Digoxigenin-labeled probes were synthesized from PCR-amplified templates, sections were mounted on 96-well plates, and hybridization followed by immunohistochemistry for the probes was performed in each well of the plates [16].

In the present paper, as a result of the screening for adult mouse testes, we found that the mRNA for *Tmco5* (*Transmembrane and coiled-coil domains 5*) is expressed in the round and elongated spermatids. Gene Database (Gene ID: 67356, https://www.ncbi.nlm.nih.gov/gene/) confirmed that the expression of the mRNA is restricted in the adult testes and that the deduced amino acid sequence has a coiled-coil domain in the N-terminal region and a transmembrane domain in the C-terminal region with the calculated molecular weight of the protein 35,850. Regarding the expression of the gene, Kwon et al. reported the expression of thirteen testis-specific genes including Tmco5 by reverse transcriptase, PCR, and immunoblotting [17]. The function of this gene has not yet been reported. However, a homolog of this gene in *Drosophila*, *Transport and Golgi organization protein 6* (*Tango6*, *Tmco7*), has been proposed to be required for the organization of Golgi apparatus. GFP-tagged Golgi apparatus is scattered when RNAi for *Tango6* is introduced in Schneider 2 cell-lines [18]. With regards to the function of the Golgi apparatus in spermatogenesis, the following two phenomena are well known. The former is the formation of the acrosome. During the process, Golgi-derived proacrosomal vesicles fuse to form and enlarge the acrosome [19]. The latter is the development of tail-structure; Golgi-derived non-acrosomal vesicles are transported to the base of the flagellum by way of the manchette, a cytoskeletal complex formed around the nucleus by a sleeve of microtubules [20]. Therefore, it is an attractive hypothesis that the function of *Tmco5* may be involved in such phenomena by regulating the organization of the Golgi apparatus.

In the present report, we first of all demonstrated the expression of *Tmco5* mRNA in the testis. Afterwards, the protein expression was determined using a monoclonal antibody raised against recombinant TMCO5 protein. Next, in order to clarify whether *Tmco5* is involved in the organization of the Golgi apparatus, GFP-tagged Golgi apparatus was observed in the CHO cells after the induction of TMCO5. Finally, in order to know whether TMCO5 is localized to the acrosome or other structures in the elongated spermatids, subcellular localization was determined. These data suggest, along with the domain structure of TMCO5, that *Tmco5* may have a role in vesicle transport along the manchette.

In addition, while we were writing the present manuscrpt, a paper on rat TMCO5 was published [21]. The experimental results of the paper were partially overlapped wtih the results we obtained, but there were some differences from ours. We will discuss this point in the later section.

## Materials and methods

### Ethics statements

All animal experiments were reviewed and approved by the Osaka City University animal subjects committee. The euthanasia of animals was done in a way that did not cause pain. Generally, pentobarbital (50 mg/ml) was intraperitoneally injected (9.1 mg/kg) and then the animals including rats and mice are euthanized by cervical dislocation.

### The high-throughput *in situ* hybridization screening

The detailed method for the screening has been reported previously [16]. Briefly, almost all the procedures were carried out by 96-well format. Digoxigenin-labeled probes were synthesized from PCR-amplified templates, sections were mounted on 96-well plates, and hybridization followed by immunohistochemistry for the probes was performed in each well of the plates.

### Recombinant protein expression and monoclonal antibody production

Partial coding region of *Tmco5* (nucleotide position from 162 to 536, NM_026104) was PCR-amplified using adult testis cDNA library with a pair of primers, 5-GGGCTAGCAAGAACATTATCAGCTTG-3 for the sense primer with a NheI site (underlined) and 5-CCAAGCTT*TTA*CAACTGTTGTAGTTTAAC-3 for the antisense primer with a stop codon (italic) as well as with a HindIII site (underlined). PrimeSTAR Max DNA Polymerase (Takara, R045A) was used, and the reaction conditions were as follows; denaturing at 98°C for 10 sec; annealing at 55°C for 5 sec; and extension at 72°C for 20 sec in 30 reaction cycles. The amplified fragment was digested with NheI and HindIII and cloned into the same sites of pRSET A vector (ThermoFisher, V35120). The obtained plasmid was transformed into BL21 (DE3) pLysS competent cells. The recombinant protein was purified using TALON Metal Affinity chromatography (GE Healthcare Life Science) according to the method of the supplier’s protocol. Female rats (Wistar) were immunized with 300 μg of the purified protein with Freund’s adjuvant three times at 2-weeks intervals. Three days after the final immunization with 300 μg of the purified protein alone, spleen cells were harvested and fused with P3U1 myeloma cells, and HAT selection (Sigma-Aldrich, H0262) was performed in RPMI-1640 medium (Sigma-Aldrich, R0883-500ML) with 10% Fetal Bovine Serum (FBS, HyClone, SH30071.03). Screening for the monoclonal antibody was performed by the conventional Enzyme-Linked Immunosorbent Assay (ELISA) using the recombinant protein described previously [22] as well as by colormetric immunohistochemistry to the sections of adult mouse testes. Positive hybridomas were further cloned by the limited dilution method, and one of the clones, termed RTm01, was used for experiments onward.

### Immunoblotting

The expression of TMCO5 protein was analyzed by SDS-PAGE and immunoblotting. Adult tissue samples including testis, ovary, skeletal muscle, brain, skin, stomach, intestine, colon, and spleen as well as developing testes of 3 to 8-weeks old mice were extracted with 5 times volume (v/w) of SDS sample buffer (5% 2-mercaptoethanol, 10% glycerol, 2% SDS, 0.005% Bromophenol Blue, and 63 mM Tris-HCl pH. 6.8) and were boiled for 5 min. After centrifugation at 17,400 x g for 10 min, supernatants were collected and 15 μl of the samples were loaded into wells of 10% SDS-PAGE gel [23]. The gel was transferred to nitrocellulose membrane [24]. Briefly washed with TBST (150 mM NaCl, and 0.1% Tween 20, and 50 mM Tris–HCl pH7.5), the membrane was treated with Blocking solution (0.5% casein in TBST) for 30 min, and then incubated for 1 h with the culture supernatant of RTm01 followed by the 3 times washing with TBST. Afterwards, 1:5,000-diluted alkaline phosphatase-conjugated anti-rat IgG antibody (BioRad, STAR131A) was incubated for 1 h. After 3 times washing with TBST for 10 min, the chromogenic reaction was performed with BCIP-NBT Solution Kit (Nacalai Tesque, 03937–60). While adopting a high-sensitivity chemiluminescent detection, we used 1:10,000-diluted horseradish peroxidase-conjugated anti-rat IgG antibody (BioRad, 5204–2504) as a second antibody, and chemiluminescence detection was conducted with ELC kit (ThermoFisher, 32106) and LAS4000min (Fujifilm).

### Colormetric immunohistochemistry

Mouse testes were fixed in Dent's fix (80% methanol and 20% dimethyl sulfoxide) for 6 h at 4°C, dehydrated in 100% ethanol, embedded in a mixture of polyester wax and cetyl alcohol (Electron Microscopy Sciences, 19312 and WAKO, 101309 respectively) at a ratio of 2:1 and sectioned to be 8 μm thick. The sections were adhered to MAS-coated glass slides (Matsunami, S091150). They were dewaxed with 100% ethanol, hydrated with TBST and blocked with the Blocking solution. The culture supernatant of RTm01 antibody was reacted for 1 h. After washing with TBST, horseradish peroxidase-conjugated anti-rat IgG (x 5,000, BioRad, 5204–2504) was incubated for 1 h. Consequently, Tmco5 protein was detected using Peroxidase Stain DAB Kit (Nacalai, 25985–50). Hematoxylin (Vector, H-3401) was used for nuclear counter-staining. After being mounted, samples were observed under a microscope.

### Cell culture

Chinese hamster ovary (CHO) cells were cultured in Dulbecco's Modified Eagle's medium (DMEM, Sigma-Aldrich, D5796-500ML) with 10% FBS (HyClone, SH30071.03).

### Generation of CHO cells, wherein Golgi apparatus is tagged with EGFP

The cDNA corresponding to the amino acid sequence from the N-terminal methionine to the position of 60 of *β-1*, *4-galactosyltransferase 1 gene* (NP_071641), where a Golgi-localization signal exists (Cole et al., 1996), was amplified with a mouse heart cDNA library as a template (sense primer 640U: 5-GATCGCTGTGGTCGGGTAG-3, antisense primer 1071L: 5-GCACTGGCAACGAAGACAAG-3). Also, the full-length coding region of EGFP was amplified (sense primer 1U: 5-ATGGTGAGCAAGGGCGAGGAG-3, antisense primer 720L with termination codon: 5-TTACTTGTACAGCTCGTCCATGC-3). Both DNA fragments were fused by the overlap extension PCR [25] and cloned into the pTA2 vector (TOYOBO). After adding HindIII and BamHI sites by PCR, the fused fragment was further cloned into the same site of eukaryotic expression vector pCAG-MCS2 [26], which was provided by Dr. Mikio Hoshino. The plasmid was transfected into CHO cells using TransIT®-LT1 Transfection Reagent (Takara, V2300), and transformants were selected in 10% FBS-DMEM with 500 μg/ml G418. EGFP-tagged Golgi-positive colonies were selected via the observation of the fluorescent distribution, which was the same as that of the CHO cells transiently transfected with CellLight Golgi-GFP BacMam 1.0 (ThermoFisher, C10592). A positive clone, termed as CHO-GolEGF, was used from here onward.

### Induced expression of Tmco5 in CHO-GolEGF

In order to introduce the inducible expression of TMCO5 in CHO-GolEGF, we utilized a Tet-on system (T-Rex System, ThermoFisher, C10592), comprising of two vectors; pcDNA TM 4/TO for the expression of the target protein and pcDNA TM 6/TR for the production of the repressor for pcDNA TM 4/TO vector. The full-length coding region of *Tmco5* (nucleotide position from 57 to 1043, NM_026104) was PCR-amplified with a pair of primers; 5-GGGGATCCCCGGATCCGCCAAAGCACATCGG-3 for sense primer with BamHI site (underlined) and 5-GGGATATCGGGATATCCTTCATCCCTCCTG-3 for antisense primer with EcoRV site (underlined). The amplified fragment was digested with BamHI and EcoRV and cloned into the same sites of pcDNA TM 4/TO. The resulting plasmid and pcDNA TM 6/TR were co-transfected into the CHO-GolEGF cells in accordance with the supplier’s protocol. For the selection of the integration of each plasmid, Zeocin (300 μg/ml) and Blasticidin (10 μl/ml) were used. One of the clones, termed as CHO-GolEGF-tmco, was selected by the immunohistochemistry using the anti-TMCO5 antibody (RTm01) after the induction of TMCO5 protein by the addition of tetracycline at the concentration of 1 μg/ml.

### Fluorescent immunohistochemistry to the CHO-GolEGF-tmco cells

CHO-GolEGF-tmco cells were grown on the surface of collagen-coated round-type coverslip (Matsunami, CO13001) in DMEM with 10% FBS including G418 (100 μg/ml), Zeocin (300 μg/ml) and Blasticidin (10 μg/ml). After 60% confluent, tetracycline was added (10 μg/ml) and another 24 h culture was performed to induce the expression of TMCO5. In the control experiment, the addition of tetracycline was omitted not to induce the expression of TMCO5. After 24 h culture, cells attached to a coverslip were washed with PBS (10x D-PBS (-), Fujifilm, 048–29805) and then fixed with 4% paraformaldehyde-PBS (Fujifilm, 161–20141) for 10 min at room temperature. After being washed with TBST, they were blocked with the Blocking solution, then reacted with culture supernatant of RTm01 antibody mixed with 1:2,000-diluted mouse anti-β-tubulin antibody (abcam, ab131205) for 1 h. After washing twice with TBST, Alexa 647-labeled anti-rat IgG (CST Japan) and Alexa 594-labeled anti-mouse IgG (CST Japan), each diluted 2,000 times with blocking solution, were reacted for 1 h. After 3 times washing with TBST for 5 min, DAPI (4′, 6-diamidino-2-phenylindole) (ThermoFisher, D1306) was used for fluorescent nuclear staining in accordance with the instruction manual. The samples were observed with a confocal microscope (TCS SP8, Leica).

### Fluorescent immunohistochemistry for sections of adult testes

The sections of mouse adult testes mounted on glass slides were reacted with culture supernatant of RTm01 antibody mixed with 1:2,000-diluted mouse anti-β-tubulin antibody (abcam, ab131205) for 1 h. After washing twice with TBST, Alexa 647-labeled anti-rat IgG (CST Japan) and Alexa 594-labeled anti-mouse IgG (CST Japan), each diluted 2,000 times with blocking solution, were reacted for 1 h. Simultaneously, for detecting acrosomes, Alexa 488-labeled Lectin PNA (diluted x 200 times, Molecular Probes) was reacted. After 3 times washing with TBST for 5 min, DAPI (4′, 6-diamidino-2-phenylindole) (ThermoFisher, D1306) was used for fluorescent nuclear staining. The samples were observed with the confocal microscope (TCS SP8, Leica).

## Results

### Expression of Tmco5 mRNA and protein

**Fig 1:**
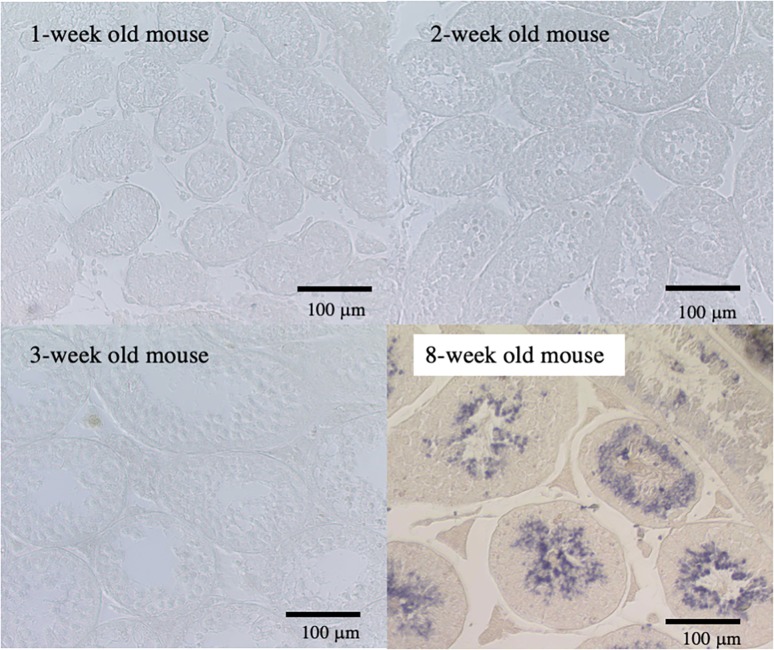
*In situ* hybridization with Tmco5 probe. The sections of testis from 1-week to 8-week old mice were hybridized with Tmco5 probe, indicating that the mRNA for *Tmco5* is expressed in the round and elongating spermatids in the seminiferous tubes of adult testis (8-week old mouse). In addition, we could not detect the expression of Tmco5 mRNA in the testis until mice were 3-weeks old. Scale bars are 100 μm.

As a result of the high-throughput *in situ* hybridization screening for adult mouse testes, the mRNA for *Tmco5* gene was found to be expressed in the round and elongated spermatids in the seminiferous tubules of the adult mouse, but it could not be detected before the testis of the 3-week old mouse as shown in Fig 1.

**Fig 2:**
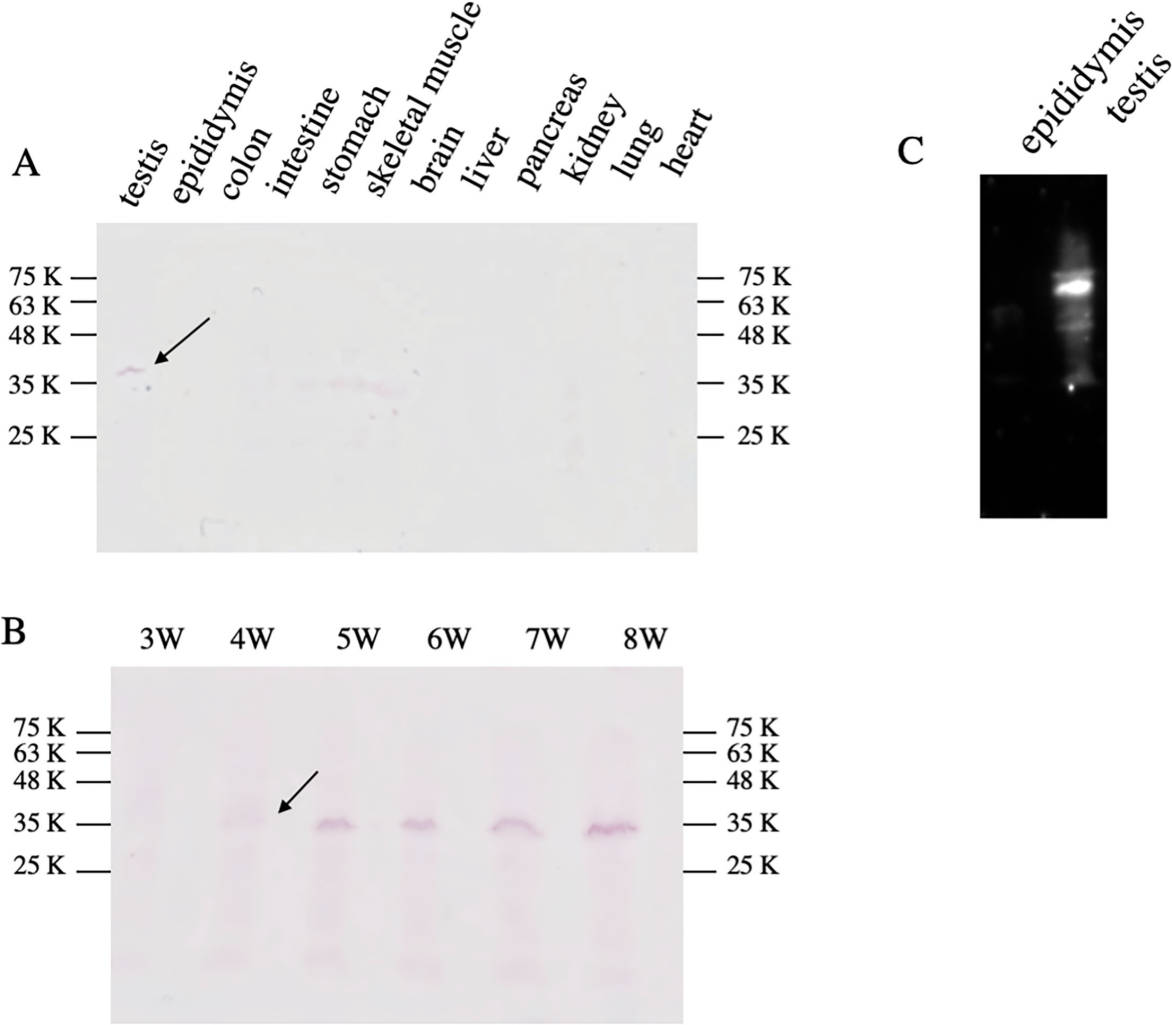
Immunobloting with the anti-TMCO5 monoclonal antibody (RTm01). (A) In the adult tissues, TMCO5 is expressed only in the testis. The arrow indicates the corresponding 36 K-band of TMCO5. Molecular weight markers are shown. (B) TMCO5 is detectable in the testis after mice were 4-weeks old. The arrow indicates the bands of TMCO5 (C) Even a highly-sensitive method using chemiluminescent detection could not detect TMCO5 in the epididymis, indicating that TMCO5 is not a component of sperm. Molecular weight markers are shown.

The expression of the protein was examined by the immunoblotting as well as the immunohistochemistry using the monoclonal antibody (RTm01) raised against the recombinant Tmco5 protein. As shown in Fig 2A, TMCO5 is specifically expressed in the testis of the adult male mouse, and the expression was detected in the testes after 4 weeks of age as shown in Fig 2B. Fig 2C shows that Tmco5 protein could not be detected in the extract of epididymis tissue, even by using the highly-sensitive chemiluminescent method, indicating that the protein is not a component of the sperm.

**Fig 3:**
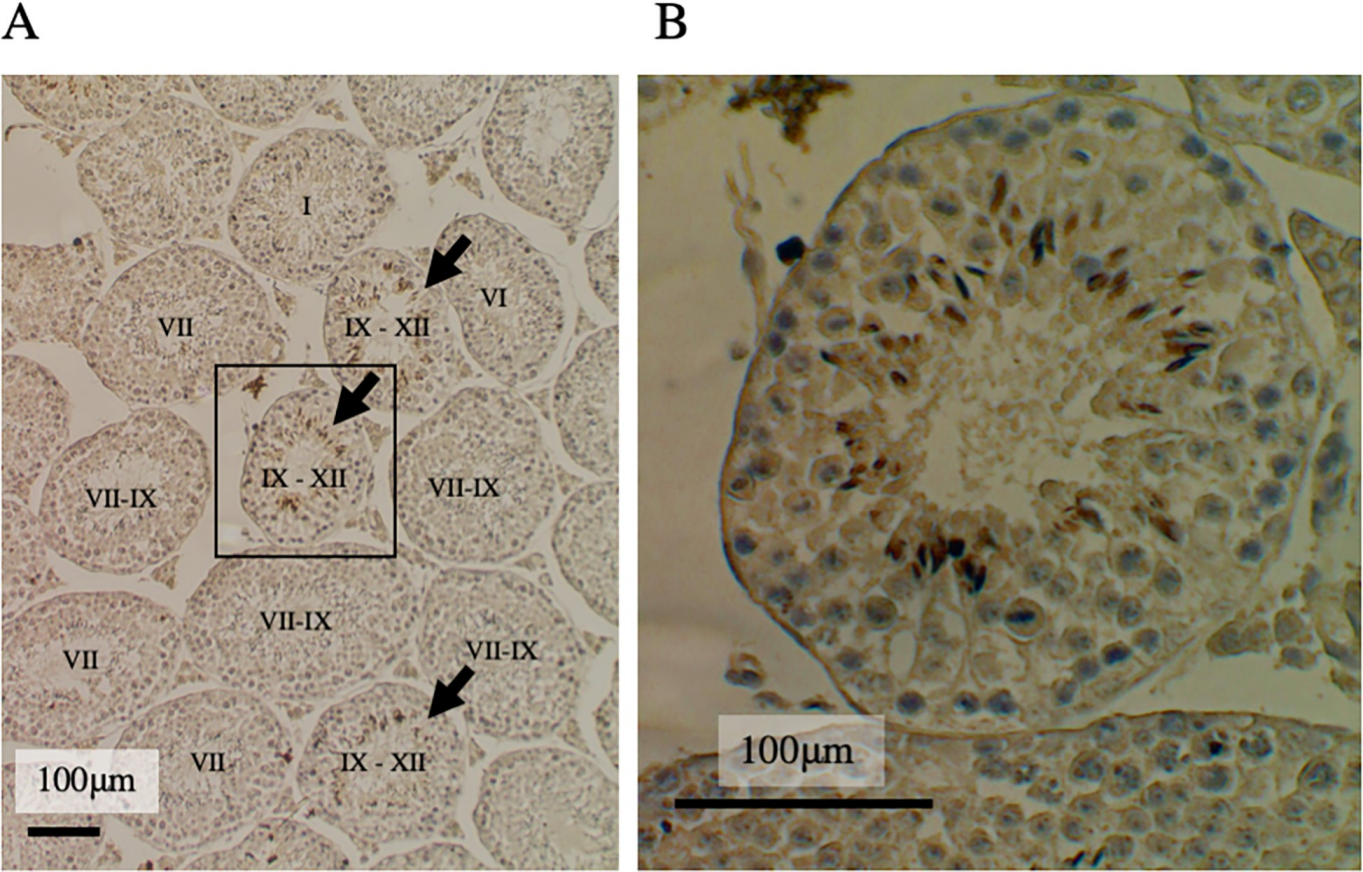
Immunostaining of adult testis using RTm01 antibody followed by nuclear counterstaining with Hematoxylin. (A) Not all the seminiferous tubes are stained, only the tubes of stage IX to XII (indicated by arrows) are stained. The indications on the seminiferous tubules show the stages of the seminiferous tubules. Scale bar is 100 μm. (B) Brown-colored positive cells in the enlarged area surrounded by the square are spermatids, indicating that TMCO5 is expressed in step 9 to 12 spermatids. Scale bar is 100 μm.

Next, in order to know when the protein is expressed during the spermiogenesis steps, enzymatic immunohistochemistry was performed, followed by Hematoxylin staining for determining the stage of the seminiferous tubules histologically considering the arrangement of the cells and the morphology of the nucleus [27] as shown in Fig 3A. Fig 3B shows that the head region of the spermatid is stained in the seminiferous tubules of stage IX to XII, indicating that Tmco5 protein is expressed in the elongating spermatids of step 9 to 12. It is well known that first spermatogenesis, named as a first-round wave, occurs soon after the birth of male mice. In this wave, spermiogenesis at step 9–12 begins around postnatal day 25 [28]. This result is consistent with that in Fig 2B, where TMCO5 is detectable in the testis after mice are 4-week old.

### Induced expression of TMCO5 protein in the CHO cells, of which the Golgi apparatus is tagged with EGFP

**Fig 4:**
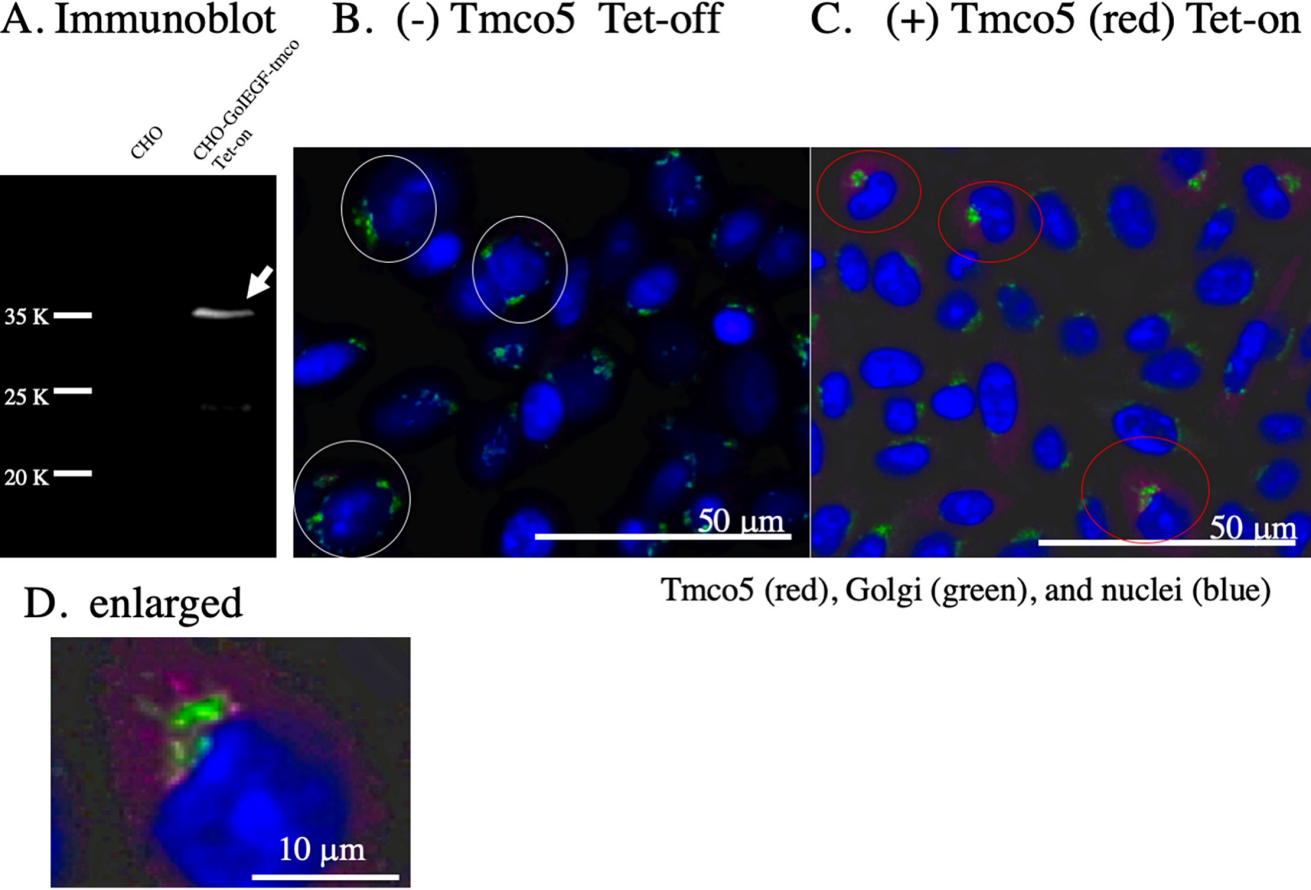
Induced expression of Tmco5 in the CHO cells whose Golgi apparatus is tagged with EGFP. (A) Immunoblotting with the anti-TMCO5 monoclonal antibody (RTm01) using the extract of CHO and TMCO5-induced CHO cell line. The arrow indicates the corresponding 36 K-band of TMCO5. Molecular weight markers are shown. (B) Without induction, green-colored Golgi apparatus is scattered around the nuclei (blue) as shown in the cells surrounded by the white circles. Scale bar is 50 μm. (C) With the induction of TMCO5 (red), Golgi apparatus (green) concentrates to the point at the center of the region, where TMCO5 is distributed, as shown in the red-colored circles. Scale bar is 50 μm. (D) The enlarged picture of the surrounded area by the red circles in C are shown. The fluorescent image of TMCO5 may be fibrous like that of the cytoskeleton. Scale bar is 10 μm.

**Fig 5:**
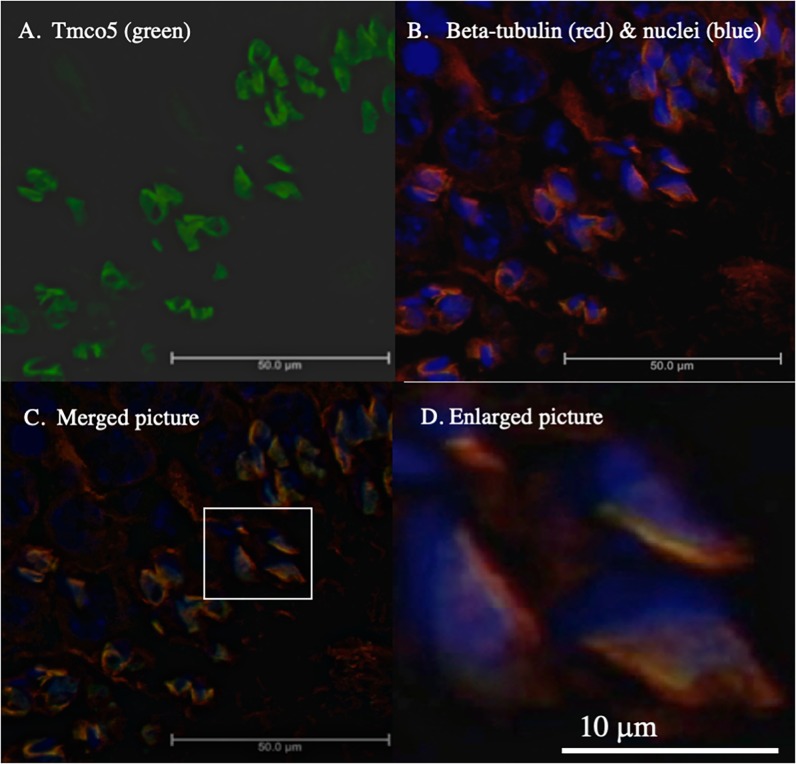
Co-localization of TMCO5 and β-tubulin in the CHO cells. The intracellular localization of TMCO5 and β-tubulin was examined in the TMCO5-induced CHO cells. Localization of TMCO5 (green) and β-tubulin (red) and nuclei (blue) was determined as shown in Fig 5A and 5B. The merged picture in C shows that both are co-localized around the nuclei (blue), suggesting that TMCO5 is a component of the microtubules or that of the vehicles moving on the network. (D) The enlarged pictures of the surrounded area by the square show that coexisting areas are not entirely identical, but the area of tubulin is slightly wider than that of TMCO5. Scale bars are 50 μm for A to C and 10 μm for D.

So far, the function of *Tmco5* has not yet been elucidated. However, it has been reported that RNAi-mediated knockdown of *Tango6* (*Tmco7*), one of the members of the Tmco-protein family in *Drosophila*, results in the fragmentation of Golgi apparatus in the S2 cell line [18]. It suggests the role of *Tango6* is in Golgi organization. Accordingly, whether *Tmco5* has a function for the organization Golgi apparatus, we conditionally induced the expression of the TMCO5 protein by using the Tet-on expression system in the CHO cell line, whose Golgi apparatus had been EGFP-tagged [29]. First, immunoblotting was performed with the RTm01 antibody using extracts of CHO cells and cells in which TMCO5 was induced. Fig 4A shows that the antibody does not recognize any proteins in the extract of CHO cells, and TMCO5 was successfully induced when tetracycline was added. As shown in Fig 4B, the Golgi apparatus was observed to be scattered around the nucleus before the induction of TMCO5. On the other hand, as shown in Fig 4C, after inducting expression, Golgi apparatus concentrated to one point at the center of the region where TMCO5 was distributed. This result coincides with that of the RNAi-mediated functional assay of *Tango6*. In addition, Fig 4D shows that the fluorescence image of TMCO5 may be fibrous like that of the cytoskeleton. We then compared the distribution of TMCO5 with that of β-tubulin in the TMCO5-induced CHO cell line. Fig 5A shows the distribution of TMCO5, and that of β-tubulin and nuclei respectively. The merged picture shown in Fig 5B indicates that TMCO5 and β-tubulin, as expected, are co-localized (the yellow-colored region). However, the region of TMCO5 is slightly thinner than that of β-tubulin as shown in the enlarged picture (Fig 5D).

### TMCO5 localizes to the manchette of the spermatids during spermiogenesis

**Fig 6:**
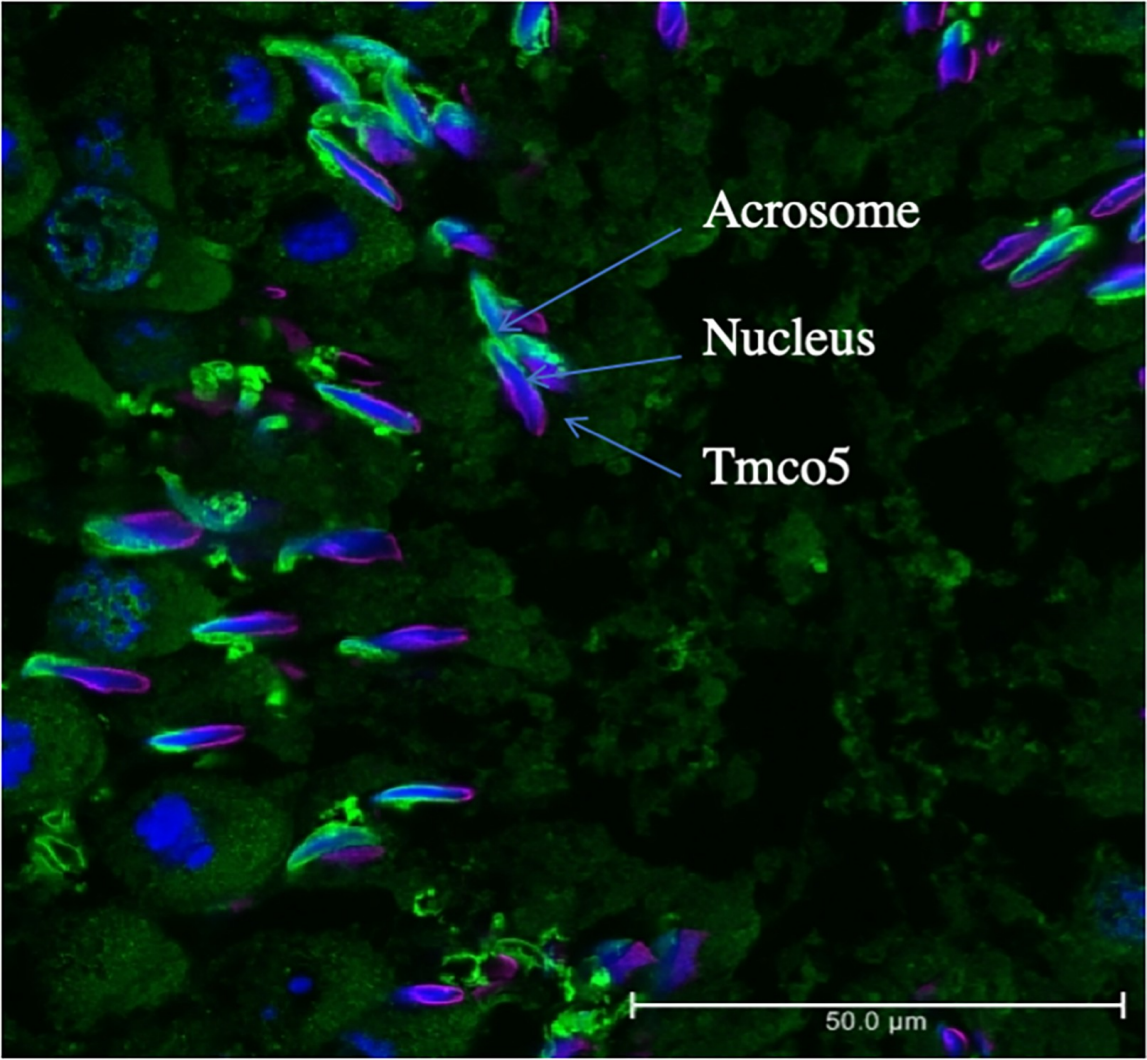
Immunofluorescent staining of adult testis. (A) TMCO5 (red) is localized to the opposite side of the acrosome (green) across the nucleus (blue), indicating that TMCO5 may be localized to the manchette. The bars indicate the position of the acrosome, nucleus, and TMCO5 positive region respectively. Scale bar is 50 μm.

**Fig 7:**
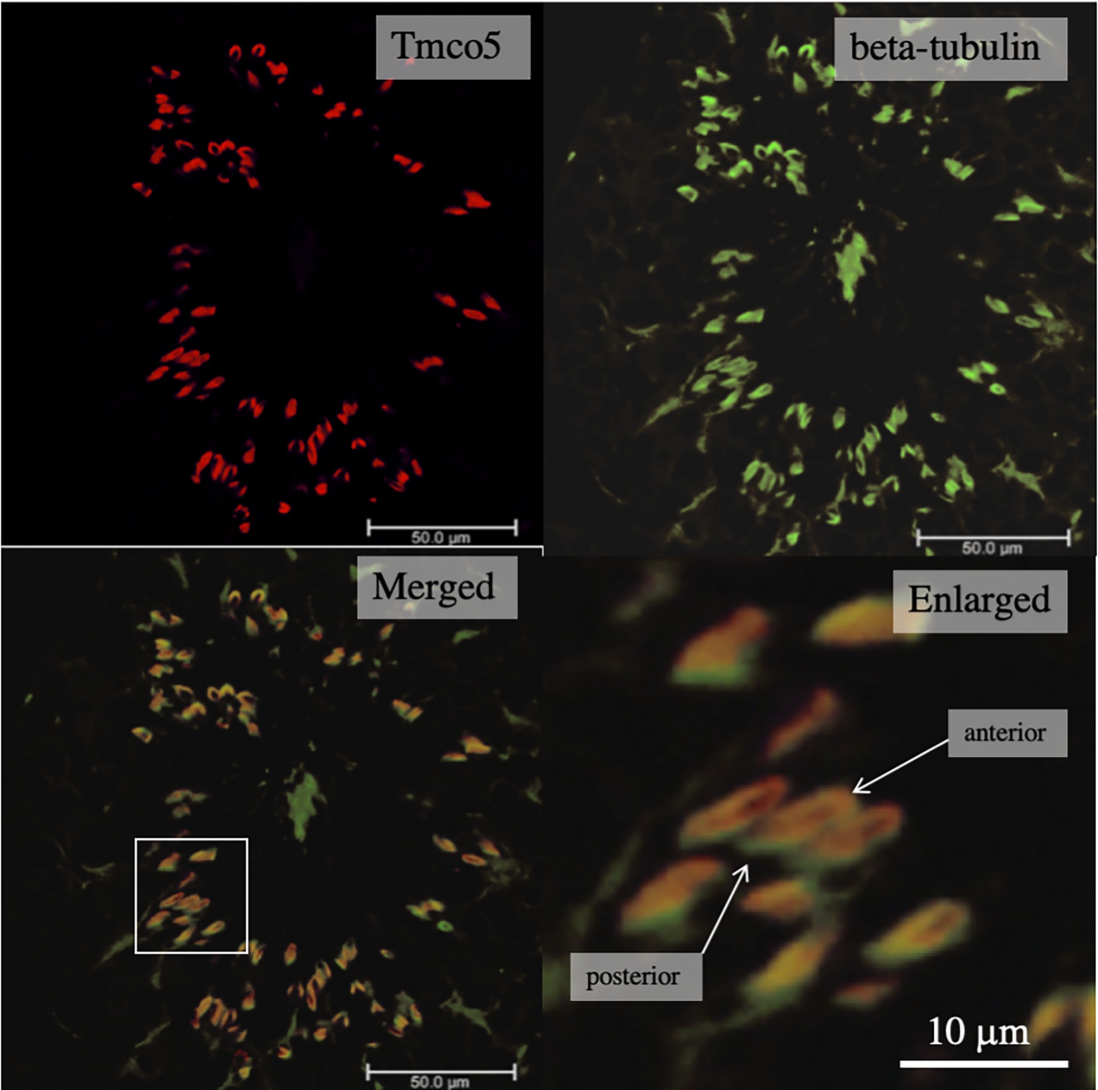
Co-localization of TMCO5 and β-tubulin in the spermatids. TMCO5 (red) and β-tubulin (green) are co-localized in the spermatids of adult testes (merged). The enlarged picture indicates that both are almost co-localized. However, in the most posterior region of the manchette, TMCO5-signal is not detectable. Scale of bars are indicated in each figure.

**Fig 8:**
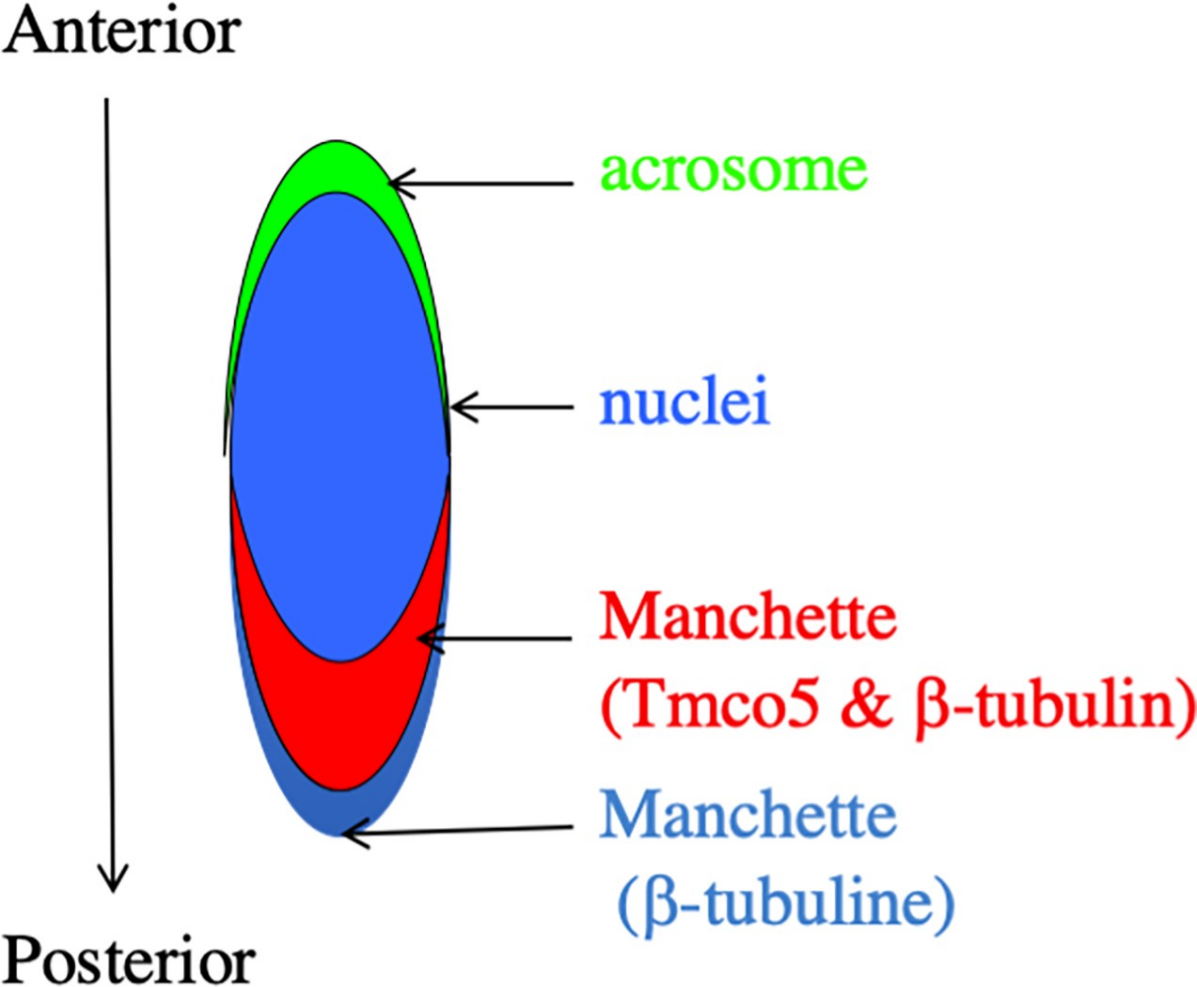
The schematic diagram of manchette. Relative position of acrosome, nucleus, TMCO5-positive, and negative manchette is illustrated according to the anterior to posterior direction.

In order to clarify the positional relationship among the region where TMCO5 is localized, the acrosome and the nucleus, we carried out immunofluorescent microscopy. Fig 6 shows that TMCO5 localizes to the opposite side of the acrosome across the nucleus. This region is known to be the manchette, a transiently emerging structure mainly comprising of the cytoskeleton of microtubules as well as actin filaments, serving in the transport of Golgi-derived non-acrosomal vesicles [20]. In addition to the experiments using the CHO cell line, we examined immunofluorescent study for identifying the localization of β-tubulin and that of TMCO5. Fig 7 shows the distribution of TMCO5 and β-tubulin. The merged picture indicates that both proteins are co-localized to the manchette. However, as in the experiment where TMCO5 was expressed in CHO cells, the localization of TMCO5 was not completely consistent with that of β-tubulin. It seems that TMCO5 is not localized in the most posterior part of the structure. A schematic diagram of the distribution of TMCO5 and β-tubulin is illustrated in Fig 8.

## Discussion

### *Tmco* family genes

In mouse and human, among seven *Tmco* family genes, named *Tmco1* to *Tmco7*, three family genes including *Tmco2*, *Tmco5* and *Tmco7* are classified into a group of having a single transmembrane in the C-terminal region (single-passed genes). *Tmco7* is ubiquitously expressed, however *Tmco2* and *Tmco5* are specifically expressed in the testis (Gene database, https://www.ncbi.nlm.nih.gov/gene/69469, https://www.ncbi.nlm.nih.gov/gene/67356). Although very little is known about the function of the three single-passed genes, *Drosophila tango6* (*Tmco7*) has been suggested to be required for the organization of the Golgi apparatus [18]. Interestingly, TMCO proteins are considered to be classified as "tail-anchored proteins", which are reported to be inserted post-translationally in the membranes of the endoplasmic reticulum, mitochondria, and peroxisome via the transmembrane domain [30, 31]. Therefore, *Tmco* family genes may be involved in membrane trafficking or vesicular transportation.

### Time-lag between the tmco5 transcription and TMCO5 translation

In the adult testis, the mRNA for *Tmco5* was detected in the round and elongating spermatids. We developed a monoclonal antibody (RTm01) against recombinant TMCO5 protein, and demonstrate that TMCO5 protein was detected only in step 9 to 12 elongating spermatids. This was confirmed by the immunoblotting analysis, in which TMCO5 could be detected in the testis of 4-week old mice. In the first wave, spermiogenesis at step 9–12 begins around postnatal day 25 [28]. The time-lag between the transcription and translation in the spermatids is well recognized. Since the structure of chromatin is condensed gradually because of exchanging the chromatin-binding proteins from histone to protamine by way of transition proteins, transcriptional activity is declined gradually in the elongating spermatids [3]. Therefore, the transcripts, such as Tmco5, which are translated after elongating spermatids for spermiogenesis, are transcribed in the round spermatids and are stored as translationally repressed state until use. With regards to this regulatory mechanism, RNA-binding proteins and corresponding cis-elements, mainly located in the 3' untranslated region, are known to be involved [32]. For example, the mRNA for *Protamin 2* is transcribed at step 7. However, it is translationally repressed afterward and is de-repressed at step 13. Both the repression and de-repression are regulated by two isoforms of CBF-A, p37 or p42, both of which are directly bind to the A2RE/RTS element in the 3' UTR of the mRNA respectively [33]. It is interesting that one cis-element is shared by two isoforms of the same gene product for generating the exact timing of the translation. In addition to the above mechanism, several lines of evidence have suggested that small RNAs including microRNAs have a role in the translational control in the testis [34, 35]. Although we could not find any cis-elements in the 3'UTR of Tmco5 mRNA using ARE site database (http://nibiru.tbi.univie.ac.at/AREsite2/welcome), four candidate miRNAs targeting the Tmco5 mRNA were identified (mmu-miR-3097-5p, mmu-miR-6539, mmu-miR-1191b-3p, mmu-miR-6368) using miRBase database (http://www.mirbase.org).

### Expression and localization of TMCO5

In our experiments, TMCO5 expression was restricted only in step 9 to 12 spermatids. However, Kaneko et al. reported that TMCO5 expression was observed not only in the elongating spermatid but also in the round and almost developed spermatids [21]. In this regard, we cannot precisely explain the reason; however, we have two possibilities. One possibility is that since the period and stage of the spermatogenesis cycle is different between mice and rats; 233.6 h versus 310.8 h for one cycle and consisting 12 versus 13 stages respectively [27], the timing of gene expression may be different. The other possibility is that the antibody used in each experiment is different. Kaneko et al. used a polyclonal antibody against synthetic oligopeptides [21]; we used a monoclonal antibody (RTm01) against a recombinant partial TMCO5A protein containing 124 amino acids. It is well known that the antibodies against oligopeptides have low specificity and are often unsuitable for immunostaining. Conversely, because monoclonal antibodies recognize restricted antigenic determinants, it is possible that the monoclonal antibody (RTm01) recognizes the three-dimensional structure of TMCO5 protein so that the antibody could not recognize the protein in the round and differentiated spermatids. However, immunoblotting analysis in Fig 2B as well as that of Kwon et al. [17] shows that TMCO5 expression starts from 4 weeks-old and 28-postnatal days, respectively, which indicates that TMCO5 expression starts from elongating spermatids in mice [17, 28, 36]. Taken together, the difference in the expression may be due to the difference in mouse and rat spermatogenesis. However, to reach a conclusion, more detailed analysis is required.

In addition, unlike our induced expression experiments using CHO cell line, where TMCO5 is localized to almost the same region as β-tubulin, Kaneko et al. reported that forced expression of TMCO5 in the COS cells resulted in the localization of TMOC5 to the endoplasmic reticulum instead of the microtubules [21]. This difference may be due to the expression system and host cell types. We used the Tet-on induced expression system with CHO cells as a permanent cell line. On the other hand, Kaneko et al. performed a transient transfection into COS-7 cells as a host cell line with a vector containing the SV40 promoter and enhancer. COS cells are known to produce large T antigen that facilitates the replication of the plasmid vectors with SV40 promoter and enhancer, which may cause a strong overexpression of target proteins [37]. In the COS-cell expression system, overexpressed TMCO5 protein may not be properly transported to vesicles and may remain in the endoplasmic reticulum.

### Possible functions of TMC5 protein

The experiments of immunoblotting and immunohistochemistry using the monoclonal antibody to the recombinant TMCO5 protein revealed that the protein is expressed only in the adult testis of step 9 to 12 elongating spermatids. During this period, the shaping of the head and delivery of proteins to the developing tail actively takes place [1]. Moreover, in the experiment of induced expression of Tmco5 in CHO cells, TMCO5 was found to be co-localized with β-tubulin. Hence, we determined the subcellular localization of TMCO5 protein in relation to that of β-tubulin in the elongating spermatids and found that TMCO5 is localized to the manchette, a transient microtubule and actin-based structure contributing to the head shaping and tail development [20, 38].

Proteins, known to be localized to the manchette, are either the components of the manchette itself or the components of the cargo or vesicle molecules moving on the manchette railroad. For example, cytoplasmic actin as well as alpha and β-tubulin constitute manchette. Vesicle-motor molecules, such as Myosin VIIa and kinesin/dynein, as well as regulatory molecules, such as Rab and MyRIP and Hook 1, are localized to the manchette [39]. Although the function of *Tmco5* has not been identified, we speculate that the function is involved in the regulation of kinesin/dynein-dependent transport of the vesicle, where TMCO5 is embedded via a transmembrane domain in the C-terminal region. Although no significant signal peptide was found by using the SignalPserver (http://www.cbs.dtu.dk/services/SignalP/) as well as the SOSUIsignal server (http://harrier.nagahama-i-bio.ac.jp/sosui/sosuisignal/sosuisignal_submit.html), we assumed that the coiled-coil domain in the N-terminal region of TMCO5 (consisting of 303 amino acids) is located outside the vesicle membrane (cytosolic face). Homology search using MOTIF Search (https://www.genome.jp/tools/motif/) revealed that there are several interesting motifs including Vac_Fusion (amino acid number (aa): 170–198), Syntaxin_2 (aa: 52–161) and Synaptobrevin (aa: 84–128), all of which are located to the N-terminal upstream region of the transmembrane domain (aa: 224–246). Vac_Fusion domain is known to play a role in the cell fusion of vaccinia virus at endosomes upon infection [40, 41]. *Syntaxin-2* and *Synaptobrevin* are the members of SNARE, a large family and a key molecule to drive fusion of membranes including vesicles [42]. The structural feature of the SNARE is that they have a coiled-coil domain and a transmembrane domain in the C-terminal region, exactly classified as tail-anchored proteins previously mentioned [43]. SignalP search revealed that like *Tmco5*, mouse *syntaxin 2* (Gene ID: 13852) and mouse synaptobrevins, *Vamp2* (Gene ID: 22318) and *Vamp3* (Gene ID: 20955) do not have notable signal peptides. Judging comprehensively, TMCO5 as well as TMCO2 and TMCO7 could be considered to be a member of SNARE [44]. Therefore, probably TMCO5 is located on the cytosolic face of the vesicle that moves along the manchette.

There are several compartments in the structure of the sperm; head region including acrosomes, equatorial region, post-equatorial region, and flagellum including the connecting piece, mid-piece, principal-piece, and end-piece [45–47]. In steps 9 to 12, elongating spermatids that limitedly express Tmco5 protein, the tail-structure is actively constructed. Recently, more than 1000 proteins associated with sperm tail structures have been identified by proteomic studies [48]. Such components packed either in the Golgi-derived non-acrosomal vesicles or in protein rafts are transported first by intra-manchette transport (IMT) to the basal body region and then to the tail compartments by intra-flagellar transport (IFT) [49, 50]. The components are correctly delivered to the destination. However, the mechanism of the detailed transport system has been poorly understood. If Tmco5 functions as a SNARE, it may have a role in the recognition of the targeting-membranes upon the transition of vesicles from IMT to IFT. This hypothesis that Tmco5 functions as a SNARE is consistent with the experimental results using the Tmco5-induced CHO cells. For example, it is reported that SNARE-mediated intracellular membrane fusion occurs using liposomes, where a SNARE is introduced [51, 52], which allows the idea that Tmco5-embedded vesicles fuse to make a large-sized Golgi apparatus after the induction of TMCO5. Otherwise, since the Golgi apparatus has been proposed to function as a microtubule organization center [49, 53], Tmco5-embedded Golgi apparatus changed its characteristics and resulted in the reconstitution of the orientation of microtubules. Thereby, the rearrangement of the Golgi apparatus may eventually occur. In addition, the absence of TMCO5 in the most posterior part of the manchette may reflect that the recognition or transfer of the vesicles from IMT to IFT has undergone on the border of the areas.

There are some questions to be solved in the future. First, in order to elucidate whether TMCO5 is embedded in vesicles, biochemical analysis such as purification of vesicles using anti-TMCO5 antibody-coupled immunoaffinity chromatography will be required. Secondly, to determine that TMCO5 functions as a SNARE, target molecules that consist of the SNARE complex should be identified by immunoprecipitation or by the two-hybrid method. Thirdly, to parse the detailed relationship between the TMCO5 protein, microtubules and Golgi apparatus, knockdown experiments using the CHO-GolEGF-tmco cell line will be effective. Lastly, in order to examine whether the *Tmco5* gene plays a crucial role in spermatogenesis, *Tmco5* knockout mice could be generated and analyzed.
